# Author Correction: SUMOylation of VEGFR2 regulates its intracellular trafficking and pathological angiogenesis

**DOI:** 10.1038/s41467-019-11659-y

**Published:** 2019-08-15

**Authors:** Huanjiao Jenny Zhou, Zhe Xu, Zongren Wang, Haifeng Zhang, Zhen W. Zhuang, Michael Simons, Wang Min

**Affiliations:** 1 Interdepartmental Program in Vascular Biology and Therapeutics, Department of Pathology, Yale University School of Medicine, 10 Amistad St., New Haven, CT 06520 USA; 2 Department of Ophthalmology, First Affiliated Hospital of Jinan University, Guangzhou, 510630 Guangdong Province P. R. China; 3 The First Affiliated Hospital, Sun Yat-sen University, Zhongshan Road II, Guangzhou, 510080 P. R. China; 4 Section of Cardiology, Department of Internal Medicine, Yale University School of Medicine, 10 Amistad St., New Haven, CT 06520 USA

**Keywords:** Protein translocation, Sumoylation, Angiogenesis

Correction to: *Nature Communications* 10.1038/s41467-018-05812-2, published online 17 August 2018.

The original version of this Article omitted from the author list the 5th author Zhen W. Zhuang, who is from the Section of Cardiology, Department of Internal Medicine, Yale University School of Medicine, 10 Amistad St., New Haven, CT 06520, USA. Consequently, the following was added to the Author Contributions: ‘Z.W.Z. performed the micro-CT angiography.’

Furthermore, the original version contained errors in Fig. 5, for which we apologize. In 5g, the first and fourth images in the upper row and the third and fourth images in the lower row were inadvertently replaced with incorrect images. The correct version of Fig. 5g is:

**Figure 1:**
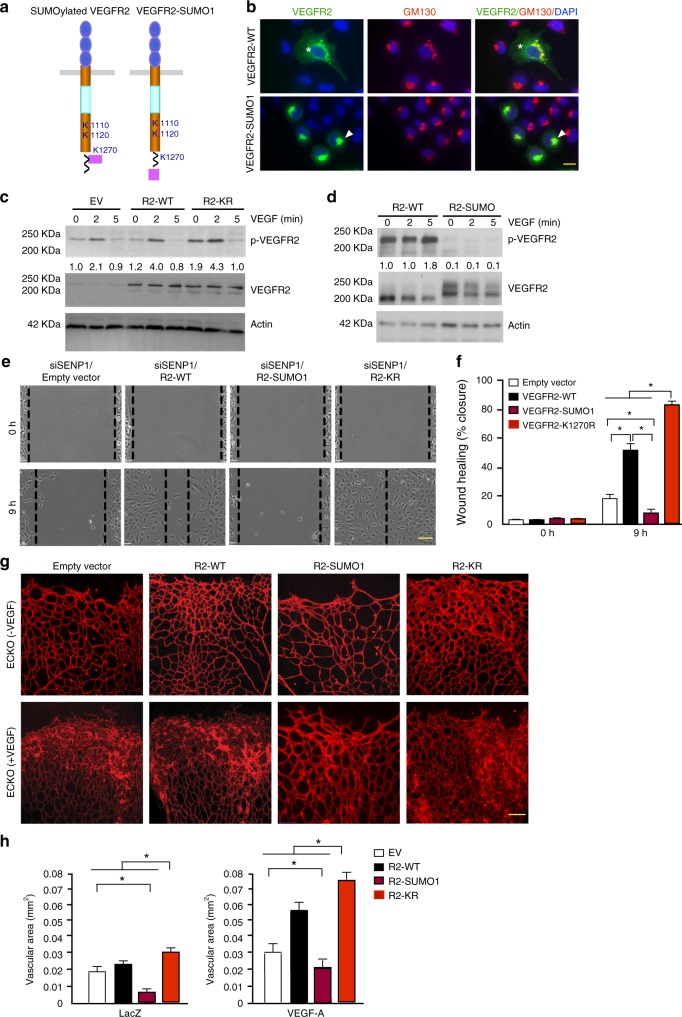
Fig. 5

which replaces the previous incorrect version:

**Figure 2:**
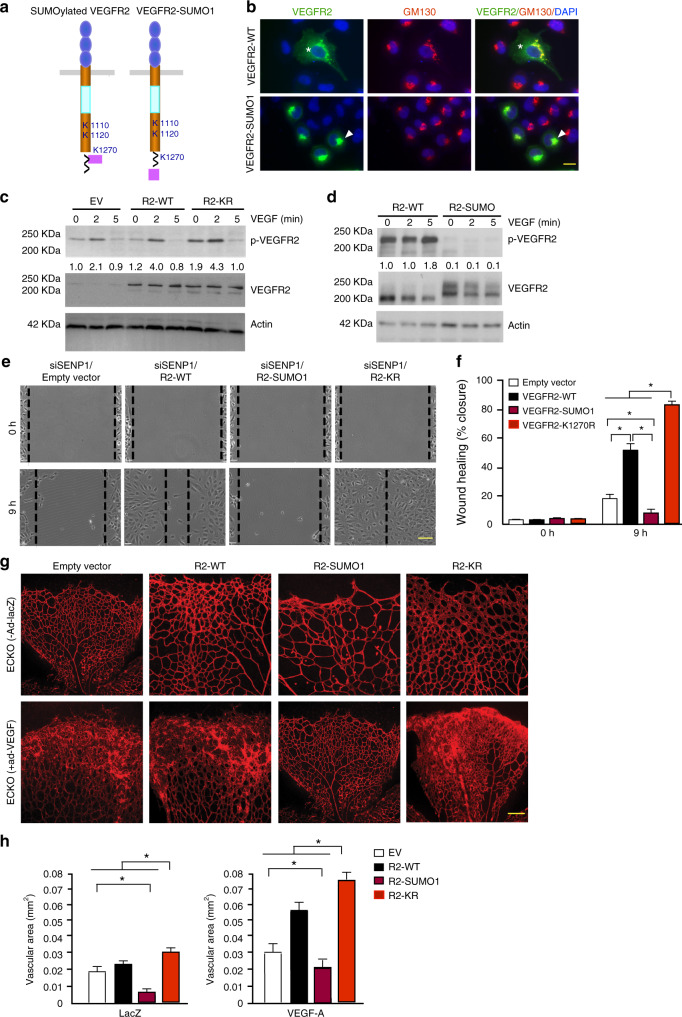
Fig. 5

The raw data associated with this experiment are provided as Supplementary Data associated with this Correction.

This has been corrected in both the PDF and HTML versions of the Article.

